# The “Mystery Cell Population” Residing in Murine Bone Marrow – A Missing Link Between Very Small Embryonic Like Stem Cells and Hematopoietic Stem Cells?

**DOI:** 10.1007/s12015-023-10581-7

**Published:** 2023-06-29

**Authors:** Kamila Bujko, Malwina Suszynska, Stephanie Franczak, Magdalena Kucia, Mariusz Z. Ratajczak, Janina Ratajczak

**Affiliations:** 1 Center for Preclinical Studies and Technology, Laboratory of Regenerative Medicine, Medical University of Warsaw, Warsaw, Poland; 2 Stem Cell Institute at Division of Hemtatology, Graham Brown Cancer Center, University of Louisville, 500 S. Floyd Street, Rm. 107, Louisville, KY 40202 USA

**Keywords:** Mystery population stem cells, VSELs, Hematopoietic specification, Markers of pluripotency, Hematopoietic reconstitution, BM stem cells

## Abstract

Bone marrow (BM) contains not only hematopoietic stem cells (HSCs) but also some very rare, early development, small quiescent stem cells that, upon activation, may differentiate across germlines. These small cells, named very small embryonic like stem cells (VSELs), can undergo specification into several types of cells including HSCs. Interestingly, murine BM is also home to a “mystery” population of small CD45^+^ stem cells with many of the phenotypic characteristics attributed to resting HSCs. Since the size of the “mystery” population cells are between that of VSELs and HSCs, and because CD45^−^ VSELs can be specified into CD45^+^ HSCs, we hypothesized that the quiescent CD45^+^ “mystery” population could be a missing developmental link between VSELs and HSCs. To support this hypothesis, we showed that VSELs first became enriched for HSCs after acquiring expression of the CD45 antigen already expressed on “mystery” stem cells. Moreover, VSELs freshly isolated from BM similar to the “mystery” population cells, are quiescent and do not reveal hematopoietic potential in in vitro and in vivo assays. However, we noticed that CD45^+^ “mystery” population cells, similar to CD45^−^ VSELs, became specified into HSCs after co-culture over OP9 stroma. We also found that mRNA for Oct-4, a pluripotency marker that is highly expressed in VSELs, is also detectable in the “mystery” population cells, albeit at a much lower level. Finally, we determined that the “mystery” population cells specified over OP9 stroma support were able to engraft and establish hematopoietic chimerism in lethally irradiated recipients. Based on these results, we propose that the murine BM “mystery” population could be an intermediate population between BM-residing VSELs and HSCs already specified for lympho-hematopoietic lineages.

**Graphical Abstract:**

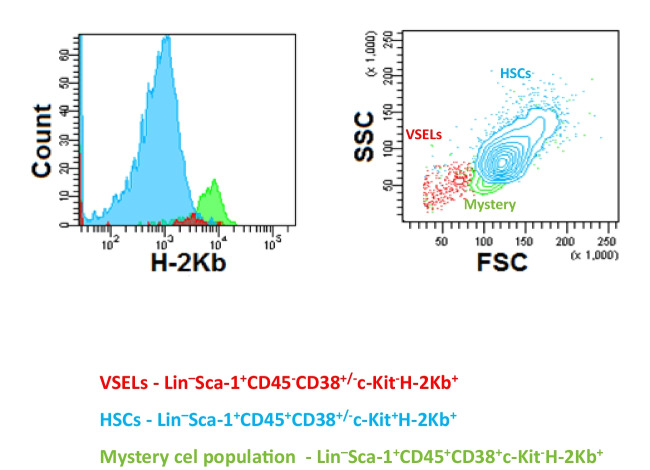

**Supplementary Information:**

The online version contains supplementary material available at 10.1007/s12015-023-10581-7.

## Introduction

More than 25 years ago, a “mystery” population of small cells with many of the phenotypic characteristics attributed to resting hematopoietic stem cells (HSCs) was identified in murine bone marrow (BM) [1]. Interestingly, these “mystery” cells express high levels of Sca-1, H-2 K, and CD38 and low levels of Thy-1.1. They are also CD45^+^ but lineage-negative (Lin^–^) for other hematopoietic markers. Furthermore, “mystery” population cells incorporate low levels of Rh123 and are resistant to the cytotoxic effects of 5-fluorouracil [1]. The only phenotypic characteristic that distinguishes these cells from Sca-1^+^, Lin^–^, CD45^+^ Thy-1.1^low^ long-term-reconstituting HSCs is the lack of c-Kit receptor expression. Nevertheless, “mystery” Sca-1^+^, Lin^–^, c-Kit, CD45^+^ stem cells freshly isolated from BM do not respond to hematopoietic growth factors in in vitro cultures, do not form in vivo spleen colonies, and do not reconstitute hematopoiesis in lethally irradiated recipient mice [1].

With our discovery in murine BM of Sca-1^+^Lin^–^CD45^–^ very small embryonic-like stem cells (VSELs) [2, 3], we became interested in this “mystery” population of potential stem cells. To justify this, VSELs, like the “mystery” population cells, are c-Kit^–^ and, if freshly isolated from BM, do not show any hematopoietic activity in standard in vitro and in vivo assays. However, quiescent CD45^–^ VSELs can be specified in OP9 stroma co-cultures into long-term reconstituting CD45^+^ HSCs [4]. Since the size of the “mystery” population is between that of VSELs and HSCs, and because these cells share a similar phenotype to VSELs (Sca-1^+^Lin^−^c-Kit^−^), we hypothesized that they could be a missing developmental intermediate between VSELs and HSCs.

To address this question, we sorted all three populations of cells from murine BM: VSELs, “mystery” population cells, and HSCs. These cells were subsequently tested in in vitro and in vivo hematopoietic assays and evaluated for expression of the Oct-4A pluripotency marker. Based on obtained data, we postulate that murine BM “mystery” cells could be an intermediate population between BM-residing VSELs and HSCs specified for lympho-hematopoietic lineages. Thus, our data sheds new light on the hierarchy of the BM stem cell compartment.

## Material and Methods

### Animals

This study was performed in accordance with the guidelines of the Animal Care and Use Committee of the University of Louisville School of Medicine and with the Guide for the Care and Use of Laboratory Animals (Department of Health and Human Services, Publication No. NIH 86–23). BM cells were isolated from sacrificed C57BL/6 mice. Congenic transplant experiments were performed by transplanting CD45.1^+^ cells into CD45.2^+^ C57BL/6 animals.

### Identification and FACS Sorting of HSCs, VSELs and “Mystery” Population Cells from Murine Bone Marrow

**Fig. 1:**
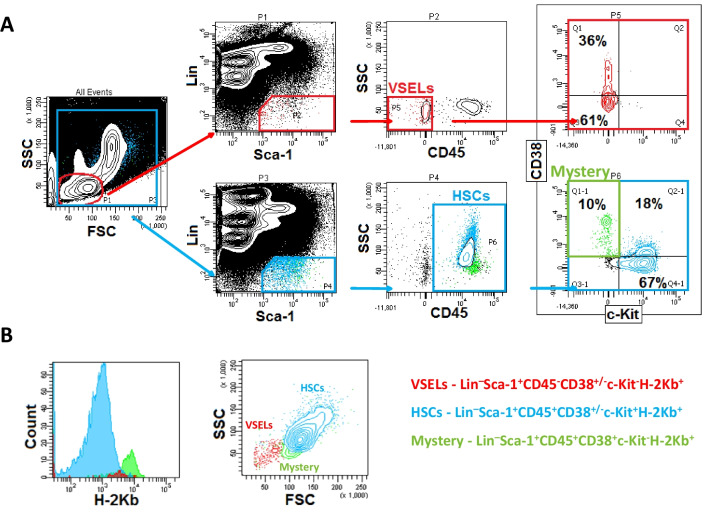
Flow cytometry analysis and gating strategy for sorting VSELs, “mystery population cells,” and HSCs. **Panel A** – Forward and side scatter properties of BM TNCs were analyzed. Specifically, small events excluding debris (P1) were further analyzed for markers specific to the VSEL population, and small events excluding debris (P3) were further analyzed for markers specific to HSCs and “mystery” population cells. Subsequently, events were analyzed for Lineage (Lin) marker and Sca-1 marker expression. Lin^−^ and Sca1^+^ events (P2 or P4) were then further analyzed for CD45 expression. Finally, CD45^−^ events (P5) were examined for CD38 and c-Kit expression and sorted as very small Lin^–^Sca-1^+^CD45^−^CD38^±^c-Kit^−^ VSELs (Q1, Q3). Simultaneously, CD45^+^ events (P6) were also analyzed for CD38 and c-Kit expression and sorted as Lin^–^Sca-1^+^CD45^+^CD38^+/–^c-Kit^+^ HSCs (Q2-1, Q4-1) and smaller Lin^–^Sca-1^+^CD45^+^CD38^+^c-Kit^−^ cells (the “mystery” population) (Q1-1). **Panel B** – the expression of H-2 Kb was analyzed in Lin^–^Sca-1^+^CD45^−^CD38^±^c-Kit^−^ VSELs, Lin^–^Sca-1^+^CD45^+^CD38^+/–^c-Kit^+^ HSCs and Lin^–^Sca-1^+^CD45^+^CD38^+^c-Kit^−^ “mystery” population cells. All sorted populations were backgated to confirm the differences in size of analyzed populations

Cells were isolated from the BM of adult male or female C57BL/6 mice (4–8 weeks old; Jackson Laboratory, Bar Harbor, ME, USA). Briefly, BM was flushed from tibias and femurs and the population of total nucleated cells (TNCs) was obtained after lysis of RBCs using 1 × BD Pharm Lyse Buffer (BD Pharmingen, San Jose, CA, USA). TNCs were subsequently stained for hematopoietic lineage markers (Lin), stem cell antigen-1 Ly-6A/E (Sca-1), CD45, CD117 (c-Kit) antigen, CD38 and MHC Class I (H-2 Kb) alloantigen for 30 min in medium containing 2% FBS. The following anti-mouse antibodies were employed for staining: Lin antibodies cocktail, all phycoerythrin (PE) rat: anti-CD45R/B220 (clone RA3-6B2), anti-Gr-1 (clone RB6-8C5), anti-TCRαβ (clone H57-597), anti-TCRγδ (clone GL3), anti-CD11b (clone M1/70), anti-Ter119 (clone TER-119) (BD Pharmingen, San Jose, CA, USA), biotin anti-Sca-1 (clone E13-161.7) with streptavidin conjugated to PE-Cy5, allophycocyanin-Cy7 (APC-Cy7) anti-CD45 (clone 30-F11) (BD Pharmingen, San Jose, CA, USA), APC anti-c-Kit (clone 2B8), fluorescein isothiocyanate (FITC) anti-CD38 (clone 90/CD38) (BD Pharmingen, San Jose, CA, USA), and Alexa Fluor 700 (AF700) anti-H-2 Kb (clone AF6-88.5) (BioLegend, San Diego, CA, USA). After incubation, cells were washed, re-suspended in RPMI 1640 medium with 2% FBS, and sorted on the MoFlo XDP cell sorter (Beckman Coulter, Brea, CA, USA) into Lin^–^Sca-1^+^CD45^+^CD38^+/–^c-Kit^+^H-2 Kb^+^ HSCs, Lin^–^Sca-1^+^CD45^+^CD38^+^c-Kit^−^H-2 Kb^+^ “mystery” population cells and Lin^–^Sca-1^+^CD45^−^CD38^±^c-Kit^−^H-2 Kb^+^ VSELs according to the gating and sorting showed in Fig. 1A and B.

### Ex vivo Differentiation of VSELs and “Mystery” Population Cells into HSCs in Primary Co-Cultures over OP9 Stromal Cells

Lin^–^Sca-1^+^CD45^+^CD38^+^c-Kit^−^H-2 Kb^+^ “mystery” population cells and Lin^–^Sca-1^+^CD45^−^CD38^±^c-Kit^−^H-2 Kb^+^ VSELs freshly sorted from murine BM were plated (10^4^) over OP9 cells in -MEM medium supplemented with 20% FBS (Molecular Probes®, Invitrogen, Waltham, MA, USA) for 5 days and subsequently trypsinized, washed by centrifugation in α-MEM, and replated in methylcellulose-based medium (StemCell Tech, Vancouver, Canada). methylcellulose cultures were solubilized and trypsinized, and the resulting cells were washed by centrifugation in α -MEM and plated into secondary methylcellulose cultures. Cells were grown in the presence of the same growth factors and replated after 5 days into new methylcellulose cultures. The same procedure was repeated for the next 2 passages.

### Evaluation of the Clonogenic Potential of Sorted Cells in Methylcellulose Cultures

VSELs, “mystery” population cells and HSCs freshly isolated from BM (10^4^), as well as cells harvested from OP9 cultures (primary cultures), were plated in methylcellulose-based medium (StemCell Tech, Vancouver, Canada) supplemented with murine stem cell growth factor (SCF), interleukin-3 (IL-3), granulocyte–macrophage colony-stimulating factor (GM-CSF), FLT3, thrombopoietin (TpO), erythropoietin (EpO), and insulin growth factor-2 (IGF-2). Cells were cultured for 5 days and the colonies formed were scored under an inverted microscope.

### PCR Analysis of Gene Expression in Freshly Sorted Cells and OP9-Expanded Cells

Total RNA from various cells (approximately 20,000 cells) was isolated using the RNeasy Mini Kit (Qiagen Inc., Valencia, CA, USA), and genomic DNA was removed using the DNA-free™ Kit (Applied Biosystems, Foster City, CA, USA). Isolated messenger RNA (mRNA) was reverse-transcribed with Taqman Reverse Transcription Reagents (Applied Biosystems, Waltham, MA, USA) according to the manufacturer’s instructions. Reverse transcription polymerase chain reaction (RT-PCR) was performed using Amplitaq Gold (Applied Biosystems, Waltham, MA, USA) and sequence-specific primers with 1 cycle of 8 min at 95 °C; 2 cycles of 2 min at 95 °C, 1 min at 62 °C, and 1 min at 72 °C; 38 cycles: 30 s at 95 °C, 1 min at 62 °C, and 1 min at 72 °C; and 1 cycle of 10 min at 72 °C. Quantitative measurement of target transcript expression was performed by q-PCR using an ABI Prism 7500 Sequence Detection System (Applied Biosystems, Waltham, MA, USA). Complementary DNA (cDNA) from indicated cells was amplified using SYBR Green PCR Master Mix (Applied Biosystems, Waltham, MA, USA) and specific primers. All primers were designed with Primer Express software (Applied Biosystems, Waltham, MA, USA), with at least one primer in each pair containing an exon–intron boundary. The threshold cycle (*C*t) was determined, and relative quantification of the expression level of target genes was obtained with the 2^–Ct^ method using -actin as an endogenous control gene and total nucleated cells (TNCs) genes as calibration controls. All primers used in PCR analysis were as follows: *Oct-4* forward primer—ACATCGCCAATCAGCTTGG, and reverse primer—AGAACCATACTCGAACCACATCC, *b-actin* forward primer – CGACGATGCTCCCCGGGCTGTA and reverse primer – CTCTTTGATGTCACGCACGATTTCCCTCT.

### Hematopoietic Transplantation Studies in Congenic Murine Transplant Model

For transplantation experiments, CD45.2^+^ mice were irradiated with a lethal dose of -irradiation (1000 cGy). After 24 h, CD45.1^+^ “mystery” population cells primed/expanded over OP9 stroma support were transplanted into mice by tail vein injection. Anesthetized, transplanted mice were sacrificed 6 weeks after transplantation to evaluate chimerism by detecting expression of transplant-derived CD45.1^+^ cells belonging to hematopoietic and lymphoid lineages in the BM of transplanted CD45.2^+^ recipients. cells were isolated from the spleens, BM, and peripheral blood (PB) of transplanted animals, and TNCs were obtained by RBC lysis using 1 × BD Pharm Lyse Buffer (BD Pharmingen, San Jose, CA, USA). TNCs were then divided and stained with FITC anti-CD45.1 (clone A20),APC anti-CD45.2 (clone 104), and one of the hematopoietic or lymphoid lineage markers: PE anti-B220 (clone RA3-6B2), PE anti-Gr-1 (clone RB6-8C5) or PE anti-CD3 (clone 145-2C11) (all BD Pharmingen, San Jose, CA, USA). The chimerism in spleen, PB and BM cells was analyzed on a BD LSR II Flow Cytometer (BD Biosciences, San Jose, CA, USA).

### Statistical Analysis

All data in gene expression analyses and colonies formation assays were analyzed using GraphPad Prism 9 (GraphPad Software, La Jolla, CA) where one-factor Analysis of Variance (ANOVA) was applied. Statistical significance was defined as *p < 0.05 or **p < 0.01.

## Results

### Cytometric Identification of VSELs, “Mystery Population Cells,” and HSCs in Murine BM

Multicolor FACS analysis was employed to compare the size and expression of surface markers between murine BM HSCs, the “mystery” cell population, and VSELs. Next, the populations of Lin^–^Sca-1^+^CD45^+^CD38^+/–^c-Kit^+^H-2 Kb^+^ cells (HSCs), smaller Lin^–^Sca-1^+^CD45^+^CD38^+^c-Kit^−^H-2 Kb^+^ cells (the “mystery” population), and very small in size Lin^–^Sca-1^+^CD45^−^CD38^±^c-Kit^−^H-2 Kb^+^ cells (VSELs) were identified and enumerated by FACS as demonstrated in Fig. 1A and B. All three cell populations were subsequently purified from BM by FACS.

### In Vitro Colony Formation by Sorted Cell Populations

All sorted cell populations (10^4^) were primed/expanded over OP9 support and subsequently evaluated for their hematopoietic potential after passaging in consecutive methylcellulose cultures. We found that, in contrast to HSCs, neither freshly sorted “mystery” BM cells nor, as expected, VSELs grew hematopoietic colonies in standard methylcellulose cultures (Supplementary Fig. 1). This step was also important in excluding contamination of sorted VSELs and “mystery” population cells with potential contaminating hematopoietic stem/progenitor cells (HSPCs).

**Fig. 2:**
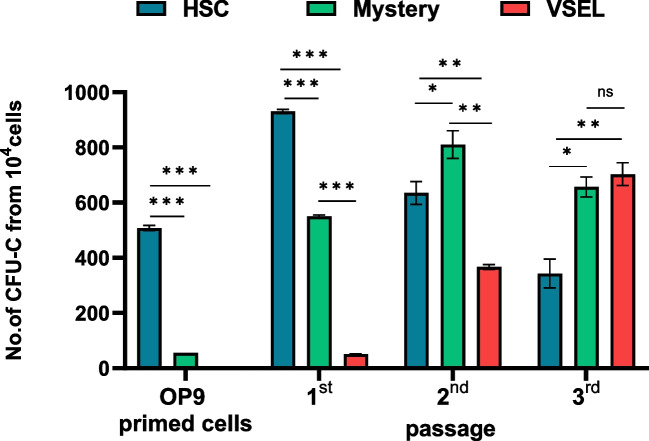
VSELs and “mystery” population cells are specified into HSCs in co-cultures over OP9 stromal cells. VSELs, HSCs, and “mystery” population cells isolated from BM by FACS were separately plated in in vitro cultures over OP-9 cell support. Subsequently cells from these primary cultures were plated in a methylcellulose-based medium supplemented with growth factors. It is shown a number of colonies formed in methylcellulose by OP9-primed/specified to hematopoietic lineage VSELs-, “mystery” population cells-, and HSCs-derived cells, and cells derived from these primary methylcellulose cultures that were replated every 5 days under the same conditions in methylcellulose (1^st^, 2^nd^ and 3.^rd^ passage). The data shown represent the combined results from three independent experiments carried out in triplicate per group (*n* = 9). ∗ *p* < 0.05; ∗  ∗ *p* < 0.001; ∗  ∗ *p < 0.0001

The most important observation was that the “mystery” population cells became specified in OP9-supported cultures into clonogenic HSPCs, and this specification occurred faster than the delayed specification of VSELs (Fig. 2). This observation supports their more advanced specification into HSPCs. VSELs first became enriched for HSPCs after gradually acquiring CD45 antigen expression.

### Expression of mRNA for Oct-4A in Murine BM-Derived VSELs, “Mystery Population Cells,” and HSCs. As Reported

**Fig. 3:**
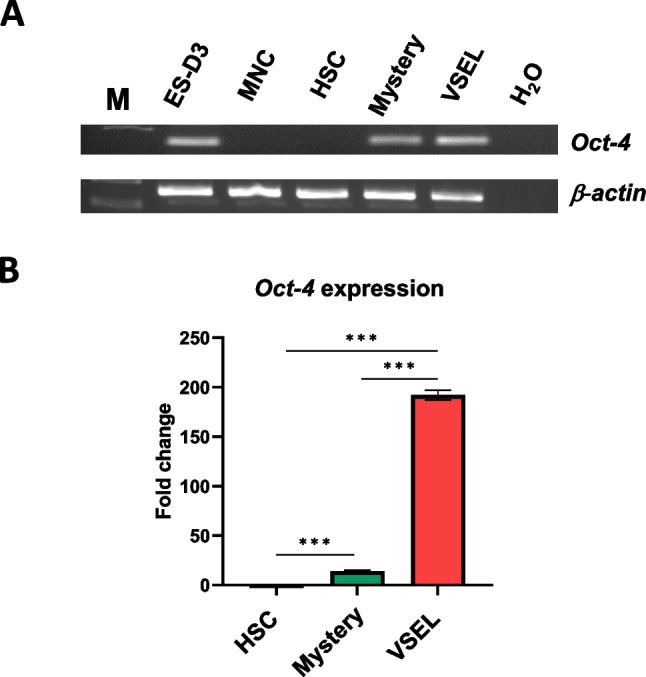
Expression of mRNA for Oct-4A. **Panel A –** RT-PCR has been performed on murine embryonic stem cell line (ES-D3), bone marrow mononuclear cells (MNC), hematopoietic stem cells (HSCs), “mystery” population cells (Mystery), and very small embryonic like stem cells (VSELs). Representative picture shown. **Panel B** – q-PCR data comparing the expression of Oct-4A in bone marrow MNC, Mystery population, and VSELs. The data shown represent the combined results from three independent experiments. ∗ *p* < 0.05; ∗  ∗ *p* < 0.001; *** *p* < 0.0001

VSELs expressseveral markers characteristic of pluripotent stem cells including the Oct-4A transcription factor. Therefore, we were interested in the expression of Oct-4A in the “mystery” cell population. We found that while VSELs showed high expression of mRNA for Oct-4A, this transcription factor was expressed at very low levels in the “mystery” cell population and was not detectable in HSCs. Figure 3A and B show RT-PCR results and q-PCR results, respectively.

### In Vivo Transplant Experiments with “Mystery” Population Cells Expanded over OP-9 Stroma Support in Congenic Murine Models

**Fig. 4:**
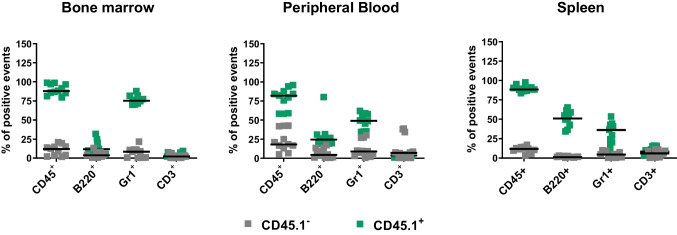
FACS analysis of chimerism in transplanted CD45.2^+^ recipients with congeneic CD45.1^+^ “mystery” population cells expanded over OP-9 stroma support. CD45.2^+^ mice were irradiated with a lethal dose of -irradiation (1000 cGy). After 24 h, CD45.1^+^ “mystery” population cells primed/expanded over OP9 stroma support were transplanted into recipient mice by tail vein. anesthetized, transplanted mice were sacrificed 6 weeks after transplantation to evaluate multilineage chimerism in the BM, PB, and spleen by detecting expression of transplant-derived CD45.1^+^ cells belonging to hematopoietic and lymphoid lineages in the BM of transplanted CD45.2^+^ recipients. The data shown represent the combined results from three independent experiments with total n = 11 experimental animals

In transplant experiments, we employed mice congenic for CD45.1 and CD45.2 expression. CD45.2^+^ mice were irradiated with a lethal dose of -irradiation (1000 cGy). After 24 h, CD45.1^+^ “mystery” population cells primed/expanded over OP9 stroma support were transplanted into recipient mice by tail vein. Anesthetized, transplanted mice were sacrificed 6 weeks after transplantation to evaluate chimerism in the BM, PB, and spleen by detecting expression of transplant-derived CD45.1^+^ cells belonging to hematopoietic and lymphoid lineages in the BM, PB and spleen of transplanted CD45.2^+^ recipients (Fig. 4).

## Discussion

The hierarchy of the BM stem cell compartment is still not defined very well. BM contains HSCs, several populations of non-hematopoietic stem cells including mesenchymal (MSCs) and endothelial progenitor cells (EPCs), as well as rare stem cells with broader, across-germ layer specification potential identified by our team and termed very small embryonic like stem cells (VSELs) [2, 3]. Although stem cells have been traditionally described as “small lymphocytes”, the exact size of various stem cell populations has been rarely measured. A recent paper postulated that small cell size is important for stem cell function in vivo, and HSC enlargement may contribute to their functional decline during aging [5, 6].

The first description of small cells with primitive morphology in murine BM was published in the context of subpopulations of the most primitive HSCs. By employing electron microscopic analysis, cells that exhibited some morphological features characteristic of hematopoietic progenitors cells were described to be between 4 and 5 µm in size [7]. At the same time, another group described a population of very primitive HSCs isolated from murine BM that were capable of long-term hematopoietic reconstitution. These cells were characterized by a lack of hematopoietic lineage markers and their ability to exclude both Rhodamine 123 (Rh^dull^) and Hoechst 33,342 (Ho^dull^) dyes. Their average size estimated by TEM analysis was again ~ 4.6 µm [8]. In another report, a population of small, long-term repopulating HSCs was isolated by employing elutriation followed by FACS sorting based on the high activity of aldehyde dehydrogenase (ALDH^high^). These cells lack hematopoietic lineage markers and were smaller than 5 µm [9–12]. cells with a similarly small size in BM have been recently described as “spore-like” SCs [13]. Furthermore, another group described a population of very small Sca-1^+^CD45^−^c-Kit^−^ cells in multiple murine tissues with the ability for multigerm layer differentiation [14].

The size of murine VSELs, identified originally by our group and confirmed by more than 50 laboratories worldwide, is ~ 4–5 mm in diameter [15]. As described, VSELs are a population of quiescent cells that can be specified into functional HSCs in co-cultures over OP9 cell stroma [4]. Of note, the same OP9 stroma support is employed for hematopoietic specification of embryonic stem cells as well as induced pluripotent stem cells [16, 17].

With our discovery of VSELs in murine BM and their potential to be specified into HSCs [4], we became interested in a small, “mystery” population of potential stem cells [1]. These cells express high levels of Sca-1, H-2 Kb, and CD38, and low levels of Thy-1.1 [1]. They are also CD45^+^ but lineage-negative (Lin^–^) for other hematopoietic markers. Furthermore, “mystery” population cells incorporate a low level of rhodamine 123 (Rh123) and are resistant to the cytotoxic effects of 5-fluorouracil [1]. Moreover, “mystery” cells freshly isolated from BM do not respond to hematopoietic growth factors in vitro, do not form in vivo spleen colonies, and do not reconstitute hematopoiesis in lethally irradiated recipient mice [1]. Interestingly, VSELs and “mystery” population cells alike are c-Kit ^–^ and, if freshly isolated from BM, do not show any hematopoietic activity in standard in vitro and in vivo assays [4]. Since the size of the “mystery” population is between that of VSELs and HSCs, we hypothesized that these cells could be a missing developmental intermediate between VSELs and HSCs.

To test this hypothesis, we purified VSELs and “mystery” population cells from murine BM. Despite the fact that “mystery” population cells freshly isolated from BM are quiescent and do not reveal in vitro and in vivo assay hematopoietic potential [1], we noticed that these “mystery” population cells, similar to VSELs, became specified into HSCs after co-culture over OP9 stroma. More importantly, “mystery” population cells specified over OP9 stroma support were able to engraft and establish multilineage hematopoietic chimerism in lethally irradiated recipients. This result supports the hypothesis that “mystery” population cells could be a population of quiescent HSCs [1]. We also found that mRNA for Oct-4, a pluripotency marker that is highly expressed in VSELs, is also detectable in “mystery” population cells, although at a much lower level [2, 3]. This expression also links the developmental origin of “mystery” population cells to VSELs. Potential contamination of highly purified from murine BM “mystery cells” and VSELs by already specified HSPCs has been excluded by a fact that these freshkly sorted cells did not form colonies in vitro (Supplementary Fig. 1).

Based on the results presented, we propose that the “mystery” cells in murine BM are an intermediate stem cell population between early development VSELs and stem cells already specified into lympho-hematopoietic lineages (HSCs).

### Supplementary Information

Below is the link to the electronic supplementary material.

Supplementary file1 (PPTX 51 KB)

## Data Availability

Detailed data available upon request.
