# Supplementary figures and images for: The “Mystery Cell Population” Residing in Murine Bone Marrow – A Missing Link Between Very Small Embryonic Like Stem Cells and Hematopoietic Stem Cells?
Source: Stem Cell Rev Rep. 2023 Jun 29;19(7):2292–8. doi: 10.1007/s12015-023-10581-7 (PMC10579127; doi:10.1007/s12015-023-10581-7)

## Slide 1
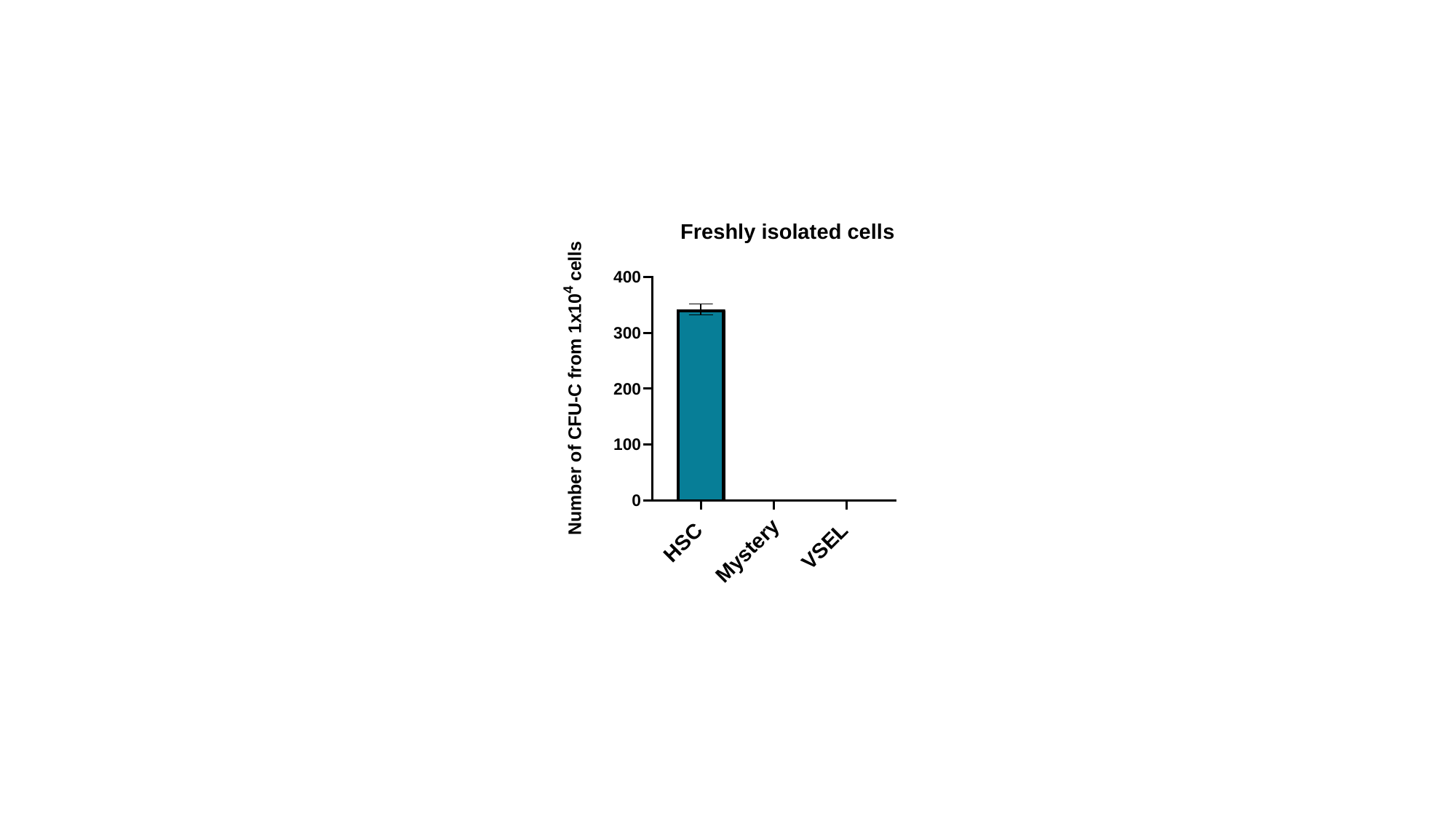

Supplement: Supplementary file 1 — Supplementary file1 (PPTX 51 KB) [file 12015_2023_10581_MOESM1_ESM.pptx]
